# Interactions of the yeast mitochondrial RNA polymerase with the +1 and +2 promoter bases dictate transcription initiation efficiency

**DOI:** 10.1093/nar/gku868

**Published:** 2014-09-23

**Authors:** Aishwarya P. Deshpande, Smita S. Patel

**Affiliations:** Department of Biochemistry and Molecular Biology, RUTGERS-Robert Wood Johnson Medical School, Piscataway, NJ 08854, USA

## Abstract

Mitochondrial promoters of *Saccharomyces cerevisiae* share a conserved −8 to +1 sequence with +1+2 AA, AG or AT initiation sequence, which dictates the efficiency of transcription initiation by the mitochondrial RNA polymerase Rpo41 and its initiation factor Mtf1. We used 2-aminopurine fluorescence to monitor promoter melting and measured the *k*_cat_/*K*_m_ of 2-mer synthesis to quantify initiation efficiency with systematic changes of the +1+2 base pairs to matched and mismatched pairs. We show that AA promoters are most efficient, followed by AG and then AT promoters, and the differences in their efficiencies stem specifically from differential melting of +1+2 region without affecting melting of the upstream −4 to −1 region. Inefficient +1+2 melting increases the initial NTPs *K*_m_s of the AG and AT promoters relative to AA or singly mispaired promoters. The 16–100-fold higher catalytic efficiency of AA initiation sequence relative to AG and AT, respectively, is partly due to Rpo41-Mtf1 interactions with the +1+2 non-template adenines that generate a stable pre-transcribing complex. We propose a model where the +2 base pair regulates the efficiency of initial transcription by controlling multiple steps including downstream promoter opening, +1+2 NTPs binding, and the rate of 2-mer synthesis.

## INTRODUCTION

The *Saccharomyces cerevisiae* mitochondrial genes are transcribed by nuclear-encoded mitochondrial RNA polymerase protein, Rpo41, which is homologous to the single subunit T7 RNA polymerase (RNAP). Unlike T7 RNAP that transcribes without requiring any initiation factors, Rpo41 requires the initiation factor Mtf1 to transcribe specifically from duplex promoters (1–6). Rpo41 by itself binds to duplex DNA with *K_d_* values of 58 nM and bends the DNA by 52°, but Rpo41 does not melt the promoter and thus cannot initiate transcription unless the promoter is pre-melted (7,8). The initiation factor Mtf1 by itself does not bind to duplex DNA (7), but it facilitates promoter melting by binding to the template and non-template strands in complex with Rpo41 (9,10). In the open complex, Rpo41 and Mtf1 bend the promoter DNA by 90° and single molecule studies show that the promoter bending and unbending is a dynamic process in the absence of initiating ribonucleotide triphosphates (NTPs) (7,11). The 2-aminopurine fluorescence studies map the melted region in the open complex from −4 to +2 and indicate efficient unstacking of the −4 and −3 base pairs (12). There are no crystal structures of mitochondrial RNAP initiation complexes, but protein–DNA crosslinking experiments have shown that Mtf1 is in proximity to the −4 to +2 region of the non-template strand and its C-terminal tail crosslinks to the −4 and −3 bases of the template strand (9,10).

The yeast mitochondrial promoter is conserved from −8 to +1, but the mitochondrial genes are transcribed with different efficiencies due to differences in the initiating sequence and in flanking upstream and downstream DNA regions (1,13–15). Previous studies have shown that the mitochondrial promoters with a purine base in the +2 non-template position are stronger than the ones with a pyrimidine (16,17). However, the biochemical basis for the preference of +1+2 AA/TT base pairs by Rpo41-Mtf1 is not understood. We used the consensus promoter sequence of the 15S rRNA that initiates with AA and designed promoter variants by systematically mutating the +1 and +2 bases of both the template and non-template strands to understand the mechanism of initiation by Rpo41-Mtf1. We measured the *k*_cat_ and *K_m_* values of the +1+2 NTPs to quantify transcription initiation efficiency in these promoter variants and used 2-aminopurine fluorescence studies to probe the formation of open complex. These quantitative studies identify the importance of the +1+2 AA promoter sequence interactions with Rpo41-Mtf1 in dictating pre-transcribing complex formation, which regulates the binding of initiating NTPs and the catalytic efficiency of transcription initiation.

## MATERIALS AND METHODS

### Proteins and oligonucleotides

Recombinant Rpo41 and Mtf1 proteins were expressed and purified as reported previously (12) using successive Ni^2+^ Sepharose 6 Fast Flow, DEAE and Heparin columns. The molar concentration of purified proteins was determined from 280 nm absorbance in Guanidium-HCl buffer and extinction coefficients (156650 M^−1^ cm^−1^ for Rpo41 and 73590 M^−1^ cm^−1^ for Mtf1).

Synthetic oligonucleotides were purchased from IDT and short 20-mer ssDNAs were HPLC purified and longer 45-mer and 57-mer were purified by urea-denatured polyacrylamide gel electrophoresis and electroelution. The molar concentration of purified DNA was determined from its extinction coefficient and absorbance at 260 nm. The double-stranded (ds) DNA were prepared by annealing complementary non-template and template strands in 1.1 to 1 ratio.

### Transcription assays

Transcription activity of Rpo41-Mtf1 on promoter DNA fragments was determined by carrying out *in vitro* transcription assays and resolving the RNA products using denaturing polyacrylamide gel electrophoresis (12). Briefly, 1 μM Rpo41, 2 μM Mtf1 and 2 μM promoter DNA fragments were incubated at 25°C in the reaction buffer containing 50 mM Tris acetate, pH 7.5, 100 mM potassium glutamate, 10 mM magnesium acetate, 0.01% protein-grade Tween 20, 1 mM DTT and 5% glycerol, and the transcription reaction was initiated by adding a mixture of 250 μM ATP, UTP, GTP and CTP (or 3’dCTP) each, spiked with [γ-^32^P]ATP or [α-^32^P]ATP. Reactions were stopped at predetermined time intervals with 400 mM ethylenediaminetetraacetic acid (EDTA) and formamide dye (98% formamide, 0.025% bromphenol blue, 10 mM EDTA), heated to 95°C for 2 min and analyzed on 24% sequencing gel containing 4 M urea. The gel was exposed to a phosphor screen overnight and scanned on a Typhoon 9410 PhosphorImager instrument (Amersham Biosciences). The free ATP and RNA bands were quantified using ImageQuant and molar amounts of RNA synthesized were calculated according to Equation (1).

(1) \begin{equation*} {\rm RNA}\,{\rm synthesized}\,(\mu {\rm M}) = \frac{{\rm R}}{{{\rm R} + {\rm A}}} \cdot [{\rm ATP}]\,(\mu {\rm M}) \end{equation*}

Where R and A are the band intensities of RNA products and free ATP, respectively, and [ATP] is the molar concentration of ATP added to the reaction. For calculation of molar amounts of RNA synthesized when using [α-^32^P]ATP, the right side of Equation (1) was divided by the number of adenines in the given length of synthesized RNA.

To determine the catalytic efficiency of 2-mer RNA synthesis, the rate of 2-mer synthesis was measured at increasing equimolar concentrations of +1 and +2 NTPs. In these transcription assays, 1 μM of Rpo41 and 2 μM Mtf1 was incubated with 2 μM of DNA at 25°C, and steady-state concentration of 2-mer RNA was measured by titrating 5–3000 μM of ATP (+ [γ-^32^P] ATP) in the case of AA promoters, or 5–3000 μM each of +1 ATP (+ [γ-^32^P] ATP) and +2 GTP or +2 UTP for AG and AU promoters, respectively. The 2-mer RNA was resolved from the free NTP on a 24% sequencing gel containing 4 M urea and quantified using ImageQuant. The rates of 2-mer RNA synthesis were calculated according to Equation (2).

(2) \begin{eqnarray*} &&{\rm Rate}\,{\rm of}\,2{\rm mer}\,{\rm RNA}\,{\rm synthesis}\,(\mu {\rm M/s})\nonumber \\ && = \frac{{{\rm R}(2)}}{{{\rm R}(2) + {\rm A}}} \cdot \frac{{[{\rm ATP}]\,(\mu {\rm M})}}{{{t}({\rm s})}} \end{eqnarray*}

Where R(2) and A are the band intensities of 2-mer RNA and free ATP, respectively, [ATP] is the molar concentration of ATP added to the reaction and *t* is the time of reaction. The rates of 2-mer RNA synthesis were plotted as a function of increasing concentration of +1 and +2 NTPs and fit to the Michaelis–Menten equation to obtain the composite *K_m_* of +1+2 NTPs and the maximum rate of 2-mer formation. Since Rpo41 was limiting at 1 μM in the Rpo41-Mtf1-DNA complex, the catalytic constant *k*_cat_ was the same as the maximum rate of 2-mer formation.

Pre-steady-state kinetic experiments were conducted at 25°C using a Model RQF-3 chemical quench-flow apparatus (KinTek Corp., Austin, TX, USA). A pre-incubated complex of 2 μM Rpo41, 2.5 μM Mtf1 and 2.5 μM U25D32 15S rRNA promoter loaded in one syringe was rapidly mixed with equal volume of 500 μM ATP from the second syringe for various time intervals, and quenched with EDTA from the third syringe. The 2-mer RNA was resolved from free ATP on a 24% sequencing gel containing 4 M urea.

### 2-Aminopurine fluorescence assay

Steady-state fluorescence measurements were carried out at 25°C on a Fluoro-Max-2 spectrofluorometer (Jobin Yvon-Spex Instruments S.A., Inc.) in buffer containing 50 mM Tris-acetate, pH 7.5, 100 mM potassium glutamate and 10 mM magnesium acetate. Fluorescence spectra of 200 nM 2-AP incorporated duplex promoters were collected from 350 to 420 nm (6-nm bandwidth) with excitation at 315 nm (2-nm bandwidth) after sequential addition of 400 nM Rpo41, 400 nM Mtf1 and 1 mM of initiating +1+2 NTPs. After subtracting contributions from buffer and proteins in the presence of unmodified DNA, the corrected 2-AP fluorescence intensities between 360 and 380 nm were integrated for comparison.

## RESULTS

### The +2 base pair dictates the transcription efficiency of the yeast mitochondrial promoters

**Figure 1.**
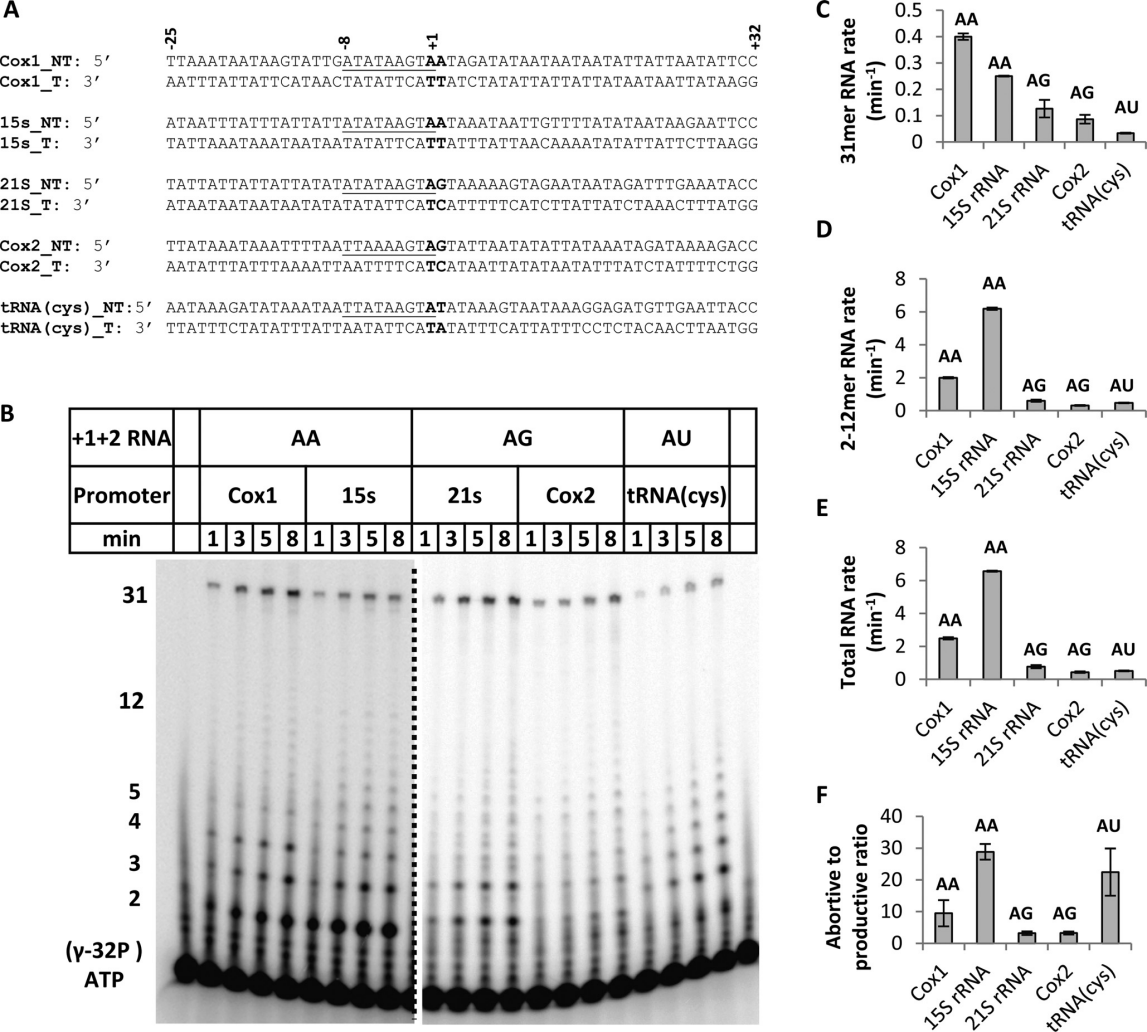
Transcription by Rpo41-Mtf1 on natural yeast mitochondrial promoters. (**A**) U25D32 fragments of the natural yeast mitochondrial promoters showing the shared nonanucleotide sequence from −8 to +1 (underlined) and different +1+2 initiation sequence in bold. (**B**) The 24% polyacrylamide-urea sequencing gel image shows the 2–12 mer abortive products and the 31-mer run-off RNA on all promoters. Transcription was carried out by pre-incubating a complex of 1 μM Rpo41, 2 μM Mtf1 and 2 μM Cox1 or 15S rRNA promoter, or a complex of 3 μM Rpo41, 4 μM Mtf1 and 4 μM 21S rRNA, Cox2 or tRNA(cys) promoter, and reacting it with 500 μM ATP (+[γ-^32^P] ATP), UTP, GTP and 3’dCTP at 25°C for 1–8 min. Lane 1, [γ-^32^P] ATP showing the impurities in that ATP batch. Lane 22, pre-quenched control sample, where 400 mM EDTA quencher was added to the reaction prior to the addition of NTPs. (**C**)–(**E**) The bands in (B) were quantified and RNA products (moles per mole of enzyme-DNA complex) as a function of time were fit to calculate the rates of RNA synthesis. Standard errors from the linear fitting of data are shown. (**F**) The ratio of 2–12 mer abortive products to productive 31-mer for each promoter. Error bars show the standard deviation from different time intervals.

All the promoters of the yeast mitochondria share a consensus sequence from −8 to −1 (underlined in Figure 1A) within its highly AT-rich genome that initiates with +1 A, but have either A:T, G:C or T:A base pair at the +2 position. The Cox1 and 15S rRNA promoters initiate with AA, the 21S rRNA and Cox2 promoters initiate with AG and tRNA(cys) promoter initiates with AU (Figure 1A). To study the efficiency of transcription initiation, we prepared DNA fragments with −25 to +32 natural sequences and quantified the *in vitro* transcription profiles of the U25D32 promoters. Purified Rpo41 plus Mtf1 is incubated with the promoter DNA and 500 μM ATP + [γ-^32^P]ATP, UTP, GTP and 3’dCTP are added to monitor RNA synthesis at 25°C from 1 to 8 min reaction times. Inclusion of 3’dCTP prevents non-templated RNA extension and a means to assess correct transcription start site. The RNA products are resolved on 24% sequencing gel to visualize the complete transcription profile (Figure 1B). Due to lesser amounts of abortive products from AG and AU promoters, we use 3-fold higher concentration of Rpo41-Mtf1-DNA complex, but for comparison, the RNA synthesis rates are normalized for protein concentration.

Visual inspection of the time course shows the expected 31-mer run-off product in all reactions indicating correct start site in all promoters (Figure 1B). Transcription on all the promoters produces abortive products from 2-mer to 8/12-mer. We observe small amounts of alternate 2–8 mer RNAs from the 21S rRNA and alternate 2-mer from Cox2 and tRNA(cys), perhaps from misincorporation of NTPs or initiation from an alternative start site. The rates of 31-mer, 2–12 mer and total RNA synthesis are calculated by plotting the molar amounts of respective RNAs (Equation (1)) as a function to reaction time and fitting it to a linear equation, where the slope of line represents the RNA synthesis rate (μM/min). To compare across various promoters, we normalize the RNA synthesis rates for protein concentration and report the rates in min^−1^. The results show that the rate of run-off RNA synthesis on the +1+2 AA Cox1 promoter is the highest at 0.4±0.01 min^−1^, followed by AA 15S rRNA promoter at 0.25±0.002 min^−1^, ∼2–3-fold lower at 0.13±0.03 min^−1^ for the 21S rRNA promoter, 0.09±0.017 min^−1^ for Cox 2 promoter, and the lowest for the AU tRNA(cys) promoter at 0.03±0.002 min^−1^ (Figure 1C). Therefore, the highest efficiency promoters are those initiating with AA, followed AG, and the least efficient is promoter initiating with AU.

The 2–12 mer abortive products are the highest on the 15S rRNA at 6.2±0.07 min^−1^, followed by Cox1 at 2±0.05 min^−1^ and ≤0.6 min^−1^ for the AG and AU promoters (Figure 1D), and the rate of abortive products correlates with the rate of total RNA synthesis (Figure 1E). The abortive to productive run-off ratio assesses the efficiency of transition from initiation to elongation. We find that Rpo41-Mtf1 undergoes ∼10 abortive events per run-off on the AA Cox1, ∼29 abortive events on the AA 15S rRNA, ∼3 abortive events on the AG 21S and Cox2 and ∼22 abortive events per run-off on the AU tRNA(cys) (Figure 1F). This ratio is mostly independent of the reaction time within 1–8 min. The results indicate that promoters that initiate with +2G or contain G in the initial coding region make less abortive products than those that are AT rich. Overall, we find that the identity of the +2 base pair in the yeast mitochondrial promoter regulates the rate of transcription initiation and the efficiency of transition into elongation.

### Rpo41-Mtf1 efficiently melts the upstream DNA region of all promoters

**Figure 2.**
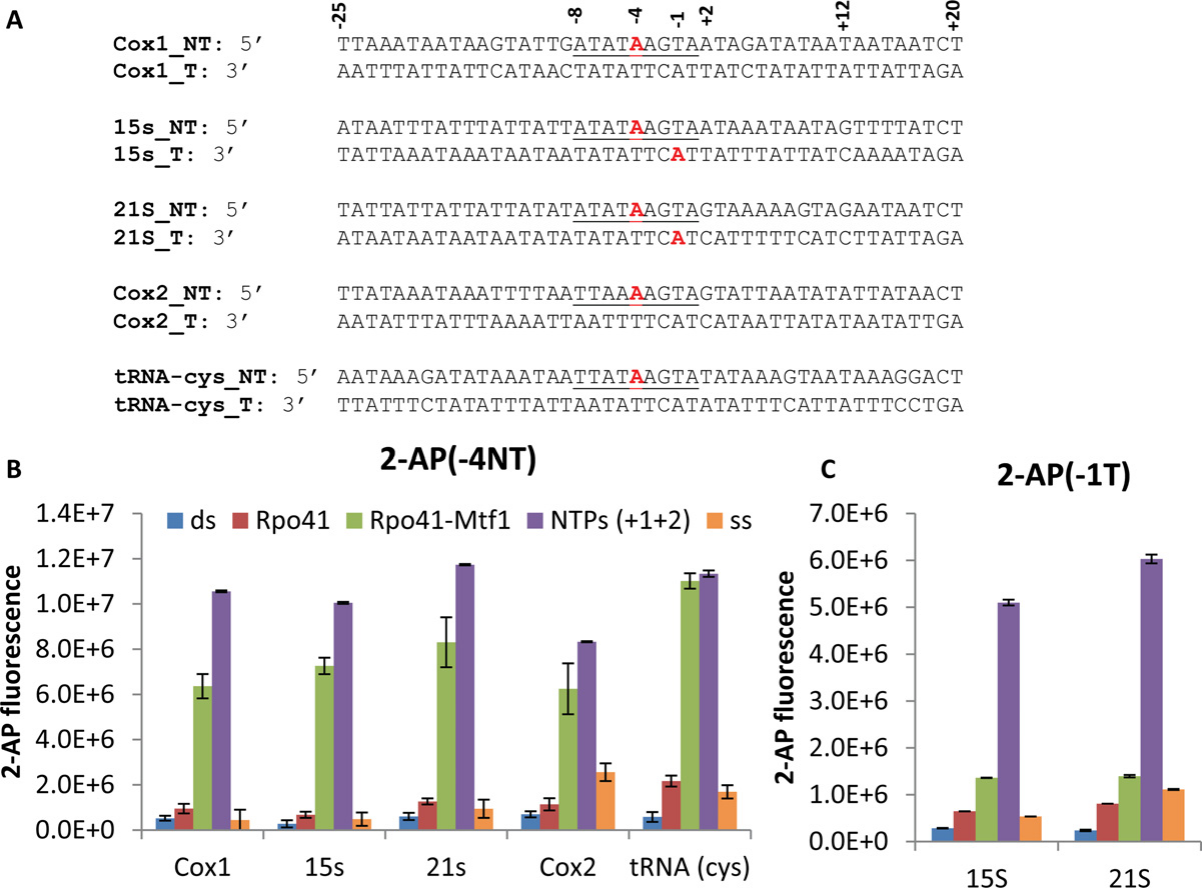
Upstream promoter melting monitored by 2-AP fluorescence studies. (**A**) The promoter U25D20 is modified with single 2-AP probe (red) either at −4 non-template or −1 template position. (**B**) Fluorescence of 2-AP in single-stranded DNA and dsDNA (200 nM) modified with 2-AP at −4 non-template. Fluorescence after sequential addition of Rpo41 and Mtf1 (400 nM each) and 1 mM of ATP to Cox1 and 15S rRNA, or ATP+GTP to 21S and Cox2 and ATP+UTP to tRNA(cys) promoter. (**C**) Similar experiments with promoter containing 2-AP at −1 position. Error bars represent standard deviation from two independent experiments (B) or from multiple measurements (C).

Previous studies have shown that the Rpo41-Mtf1 melts the +1+2 AA promoter from −4 to +2 (12). We asked whether the +1+2 sequence dictates the efficiency of promoter melting. The −25 to +20 fragments of natural promoters were labeled with a single 2-aminopurine (2-AP) fluorescent base either at the −4 non-template or the −1 template position (Figure 2A). In all promoters, the fluorescence of 2-AP in duplex DNA itself is very low (blue bar, Figure 2B), and increases slightly upon addition of Rpo41 (red bar), but the biggest increase is observed when both Rpo41 and Mtf1 are present (green bar). Addition of +1+2 NTPs further increases the 2-AP fluorescence at −4 by ∼1.4-fold on an average (purple bar). However, when we compare the final fluorescence increase, there is no significant difference in the AA versus AG or AU promoters (Figure 2B). This indicates that Rpo41-Mtf1 can efficiently unstack the −4 non-template base in all natural promoters irrespective of their +1+2 sequence.

Compared to the average 16-fold increase in 2-AP fluorescence at −4 on addition of Rpo41-Mtf1, the change in 2-AP fluorescence at the −1 position is relatively small (Figure 2C). Rpo41-Mtf1 binding to AA and AG promoters induces ∼5-fold increase in 2-AP fluorescence at −1 (green bar, Figure 2C). However, addition of ATP to 15S and ATP+GTP to 21S promoters resulted in further 4-fold increase in 2-AP fluorescence at −1 position (purple bar, Figure 2C). This indicates that binding of +1+2 NTPs efficiently unstacks the −1 template base. The results indicate that the unstacking of the −4 and −1 base pairs and hence the upstream promoter region occurs equally well in all promoters, irrespective of the +1+2 sequence. Therefore, the difference in the transcription initiation efficiencies between AA, AG and AU promoters is not due to a defect in upstream promoter melting.

### Transcription initiation efficiencies of AA, AG and AT promoters measured using 2-mer synthesis kinetics

**Figure 3.**
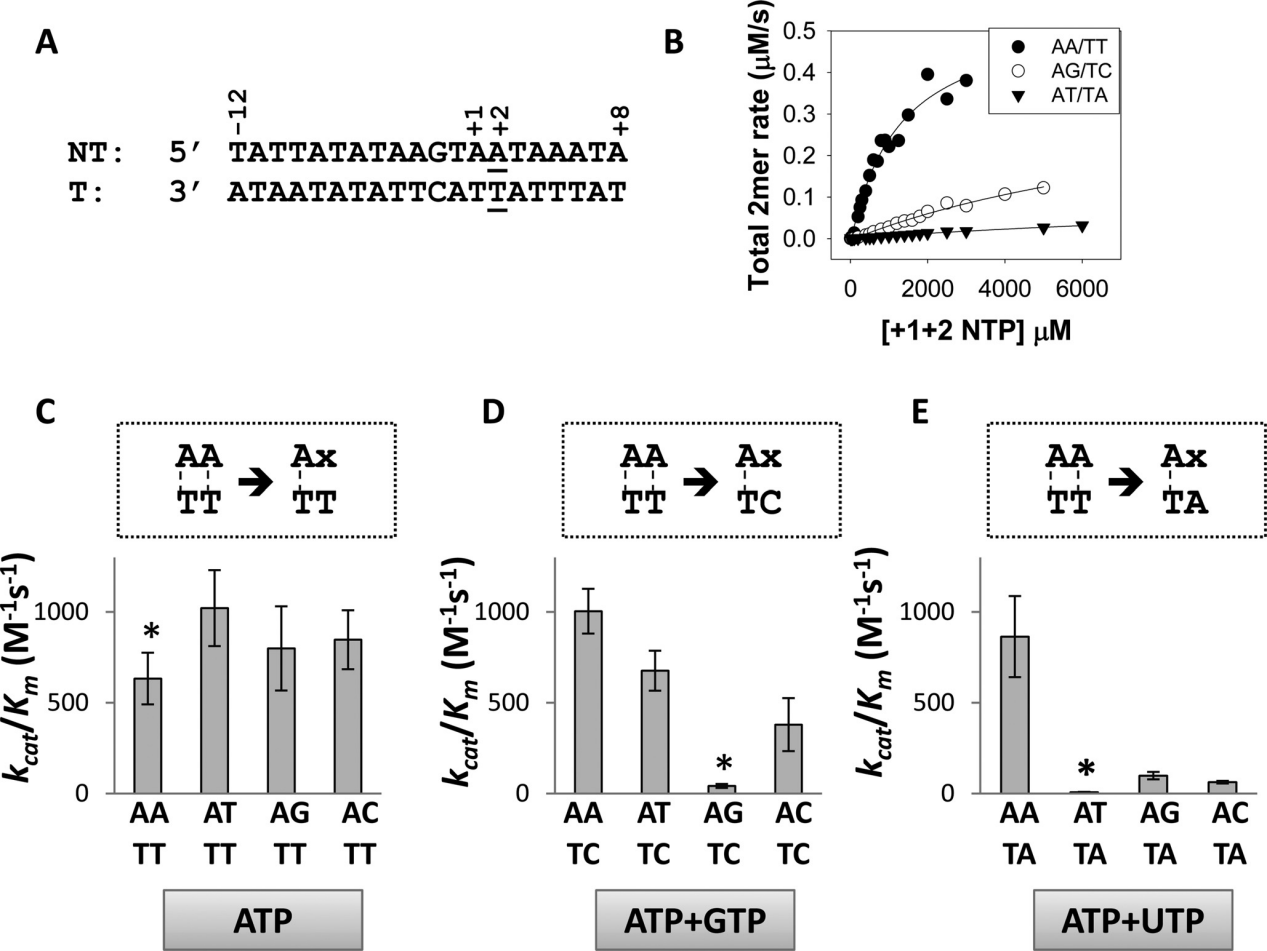
Catalytic efficiency (*k*_cat_/*K_m_*) of transcription initiation on +2 matched and mismatched pairs by Rpo41-Mtf1. (**A**) U12D8 fragment of the 15S rRNA promoter with +2 base-pair underlined. (**B**) Rates of 2-mer synthesis on AA/TT and AG/TC promoters or 2–8 mer synthesis on AT/TA promoter as a function of increasing +1 and +2 NTPs concentration fit to the Michaelis–Menten equation to obtain the *K_m_* and *k*_cat_ values. The *k*_cat_/*K*_m_ ratios of base-paired promoters (marked with an asterisk) and +2 mismatched promoters, with template sequence TT (**C**), TC (**D**) and TA (**E**) are compared. The respective initiating +1 and +2 initiating NTPs are shown at the bottom for each promoter set. Refer to Supplementary Figure S2 for detailed experimental conditions and Michaelis–Menten fittings of 2-mer rates.

The preference of Rpo41-Mtf1 to initiate on AA and AG promoters compared to AT promoters can arise from specific interactions with +1 and +2 purines in the non-template strand, +1 and +2 pyrimidines in the template strand, or initiating purine NTPs, or a combination of these factors. To understand the biochemical basis for the different transcription initiation efficiencies of +1+2 AA, AG and AT promoters, we decided to use the short 20 bp promoter DNA (U12D8) with the consensus sequence of the 15s rRNA promoter from −12 to +8 (Figure 3A), and carry out a systematic study where the +1+2 base pairs are individually changed to combinations of matched and mismatched pairs. The best parameter to compare the transcription initiation efficiencies from various promoters is the catalytic efficiency (*k*_cat_/*K*_m_) of 2-mer synthesis, which takes into consideration both the binding efficiency of the initial NTPs and the rate of 2-mer synthesis. The *k*_cat_ and *K*_m_ kinetic parameters are typically determined from the hyperbolic increase of steady-state 2-mer synthesis rates with increasing +1+2 NTPs concentrations. In order for the steady-state *k*_cat_/*K*_m_ to accurately represent the efficiency of transcription, it is important that observed rate is not limited by 2-mer RNA dissociation. We verified this with the AA and AG promoters by conducting presteady-state experiments (Supplementary Figure S1A and B). The lack of presteady-state burst of 2-mer synthesis indicates that 2-mer dissociation is faster than its synthesis rate. Hence, production of 2-mer is limited either by the chemical step or steps before synthesis such as promoter melting. Previous studies have shown that the 20 bp 15S rRNA promoter binds one molecule of Rpo41-Mtf1 to form a complex with *K*_d_ of 0.2 nM (7), and since we use saturating concentrations of Rpo41-Mtf1 (1 μM) and DNA (2 μM) and preincubate the two before addition of NTPs, promoter binding should not be rate limiting under our assay conditions. Also note that the *k*_cat_ value is the same as the maximal reaction rate in our assays since we divide it by the limiting 1 μM Rpo41 concentration of the Rpo41-Mtf1-DNA complex. Thus, monitoring 2-mer synthesis as a function of initial NTPs concentrations should provide reliable *K*_m_ and *k*_cat_ parameters that report on steps between promoter binding and 2-mer synthesis that are sensitive to NTP concentrations.

Note that the initial NTPs *K*_m_ of Rpo41-Mtf1 in the literature, measured from steady-state rates of run-off RNA synthesis as a function of initial NTPs, are much lower than what we report here (18–20). Monitoring run-off synthesis fails to provide accurate initial NTPs *K*_m_ because the initial NTPs binding step is separated from the run-off synthesis by at least as many steps as the length of the run-off product, and is rate-limited in most cases by RNA or RNAP dissociation/recycling steps. Moreover, these previous studies did not determine the *k*_cat_ values; hence, we provide the first report of the catalytic efficiency (*k*_cat_/*K*_m_) of transcription initiation from various yeast mitochondrial promoters.

The 2-mer synthesis kinetics with the AA promoter was linear over a range of ATP concentrations (Supplementary Figure S1C and D). Therefore, we measured 2-mer synthesis after 3 min reaction time as a function of increasing +1+2 NTPs concentration to estimate the *k*_cat_ and *K*_m_ values (Supplementary Figure S2A). We assure that the observed rate from the 3 min assay (0.075 μM s^−1^ at 250 μM ATP) is consistent with the observed rate from the time course (0.081 μM s^−1^ at 250 μM ATP) (Supplementary Figure S1B and Figure 3B). The AA promoter has a composite *K*_m_ of 790 μM for the initiating ATPs, and changing the +2 A:T to G:C increases the composite *K*_m_ of the initiating ATP+GTP by 10-fold to 7930 μM (Figure 3B and Table 1). Similarly, 2-mer synthesis is faster on the AA promoter with *k*_cat_ of 0.5 s^−1^ which decreases to 0.3 s^−1^ on the AG promoter (Figure 3B and Table 1). Thus, the catalytic efficiency of 2-mer synthesis or the *k*_cat_/*K*_m_ reduces by 16-fold upon changing +1+2 AA (630 M^−1^ s^−1^) to AG (40 M^−1^ s^−1^) (Figure 3C and D, asterisk).

**Table 1.** Kinetic parameters of 2-mer synthesis on promoters with +2 matched and mismatched pairs

	*k*_cat_ (s^-1^)	*K_m_* (μM)	*k*_cat_/*K_m_* (M^-1^s^-1^)	Fold increase compared to duplex DNA
**AA promoters**				
AA/TT *	0.5 ± 0.04	790 ± 166	630 ± 140	1
AT/TT	0.5 ± 0.03	480 ± 94	1040 ± 210	1.7
AG/TT	0.4 ± 0.03	510 ± 144	780 ± 230	1.2
AC/TT	0.5 ± 0.03	610 ± 112	820 ± 160	1.3
CA/TT	0.6 ± 0.04	360 ± 89	1670 ± 430	2.7
**AG promoters**				
AA/TC	0.8 ± 0.02	770 ± 92	1040 ± 120	26
AT/TC	0.4 ± 0.01	550 ± 88	730 ± 110	18
AG/TC *	0.3 ± 0.05	7930 ± 1649	40 ± 10.7	1
AC/TC	0.6 ± 0.07	1580 ± 581	380 ± 150	10
CG/TC	0.5 ± 0.05	4390 ± 957	110 ± 27	2.8
**AU promoters**				
AA/TA	0.3 ± 0.02	380 ± 96	790 ± 220	132
AT/TA *	0.1 ± 0.02	16040 ± 2975	6 ± 2	1
AG/TA	0.1 ± 0.006	1020 ± 205	100 ± 20	17
AC/TA	0.1 ± 0.004	1580 ± 177	60 ± 7	10

Promoters are classified based on initiation with +1+2 AA, AG or AU. Promoters base-paired at both +1 and +2 are marked with an asterisk. Note that the *K_m_* and *k*_cat_ parameters for AA and AG promoters are obtained from 2-mer synthesis assays, while those for AU promoters from 2–8-mer synthesis assays. Errors represent the standard error of fitting data from one to five independent experiments for different promoters. Fold change in the catalytic efficiency of 2-mer or 2–8-mer synthesis on pre-melting the DNA is shown relative to the base-paired DNA in the same class.

**Figure 4.**
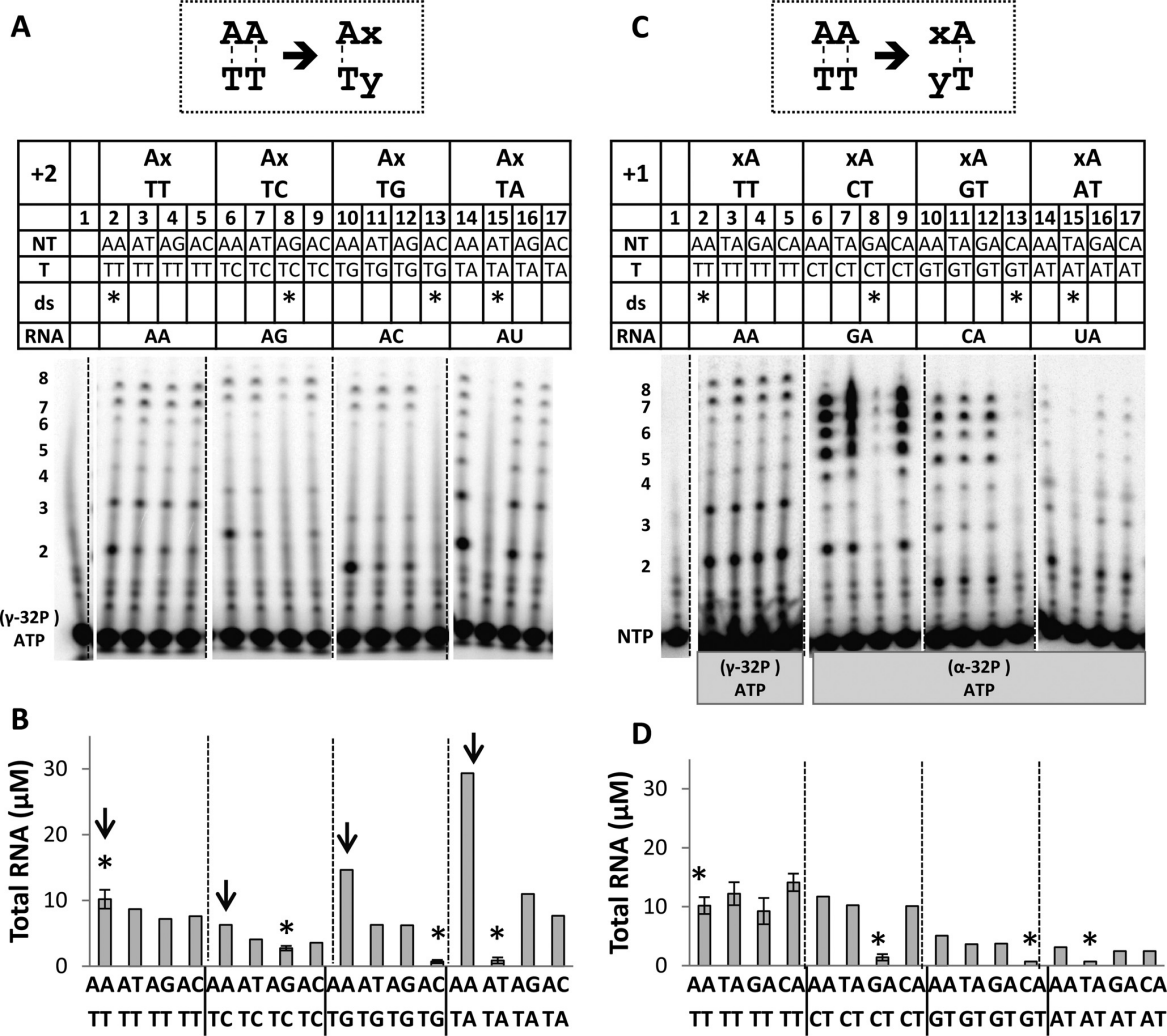
Promoter scan of changing +1 and +2 template and non-template bases by Rpo41-Mtf1. (**A**) Transcription scan of promoters with changing +2 template and non-template bases. The 24% polyacrylamide-urea sequencing gel image shows the products from 2-mer to 8-mer RNA. Promoters are grouped based on template sequence (TT, TC, TG and TA). Promoters base-paired at both +1 and +2 are marked with an asterisk. Transcription activity was measured using Rpo41 (1 μM) and Mtf1 (2 μM) on U12D8 ds DNA (2 μM) in the presence of 250 μM ATP (+[γ-^32^P] ATP), UTP, GTP and CTP at 25°C for 3 min. Lane 1, [γ-^32^P] ATP alone. (**B**) Molar amounts of total RNA plotted against +2 changes in promoters. Those with an adenine base at +2 non-template position are indicated by an arrow. (**C**) Transcription scan of promoters with changes at the +1 template and non-template bases. Transcription was carried out under conditions mentioned in (A), except [α-^32^P] ATP used to spike reactions for promoters initiating with +1 GTP, CTP or UTP (lanes 6–17). Lane 1 has the control sample with [α-^32^P] ATP alone. (**D**) Molar amounts of total RNA plotted against +1 changes in promoters.

When we mutate +2 A:T to T:A, addition of ATP+UTP results in the synthesis of run-off 8-mer, since the DNA downstream of +2 T:A promoter is 100% AT. We therefore measure total RNA synthesis (2-mer plus 3–8 mer) to determine the kinetic parameters. It should be noted that the rate of dissociation of 2–8 mer RNA can be lower than that of 2-mer RNA, which might result in a decrease in the overall 2–8 mer catalytic rate and subsequently an apparent decrease in NTPs *K*_m_. However, on comparing rates from 2-mer (Figure 3B) versus 2–8-mer (Figure 4B) assays at 250 μM NTPs, we find that on the AA/TT promoter, Rpo41-Mtf1 synthesizes 2-mer at 0.075 μM/s and 2–8-mer at a slightly lower rate of 0.055 μM/s, while on the AG/TC promoter, the 2-mer (0.01 μM/s) and 2–8-mer (0.015 μM/s) rates are comparable. This suggests that even for the AT/TA promoter, the rate of 2–8-mer synthesis, if at all, would be only slightly lower than the rate of 2-mer synthesis. Moreover, even if we cannot directly compare the rates and *K*_m_ values from 2-mer versus 2–8 mer assays, the *k*_cat_/*K*_m_ parameter should remain constant and hence comparable across promoters. Changing the +2 A:T to T:A drastically reduces *k*_cat_/*K*_m_ by 100-fold (Figure 3C and E, asterisk). This decrease in catalytic efficiency of +2 T:A promoter is mainly due to the increased NTPs *K*_m_ (Table 1), which would be even higher if were able to solely monitor the AU 2-mer synthesis. In fact, with both AG and AT promoters, the lower transcription initiation efficiency is due to higher *K*_m_ of the initiating NTPs.

### Transcription initiation efficiency is dictated by +2 base-pair melting

To investigate whether the higher *K*_m_ of the initiating NTPs with AG and AT promoters is due to inefficient promoter opening, we introduced mismatches at +2, while keeping the +1 A:T constant. The identity of the +2 non-template and template bases can influence the efficiency of promoter opening/stabilization; therefore, we changed the +2 base pair to all combinations of mismatches (Figure 3). Remarkably, changing the +2 G:C to A/C mismatch decreases the NTPs *K*_m_ by 10-fold (770 μM for +2 A/C versus 7930 μM for +2 G:C), so now the +2 mismatched promoter has almost the same NTPs *K*_m_ as the strong AA promoter (Table 1, +2 G:C marked by asterisk and Supplementary Figure S2C). The +2 A/C promoter also has higher *k*_cat_ of 2-mer synthesis (0.8 s^−1^ for +2 A/C versus 0.3 s^−1^ for +2 G:C and 0.5 s^−1^ for +2 A:T). Thus, introducing an A/C mismatch at +2 increases the catalytic efficiency by 26-fold. Changing the +2 G to other bases to create T/C and C/C mismatches increases the catalytic efficiency of 2-mer synthesis by 10–18-fold (Figure 3D). These results indicate that (i) inefficient transcription of the AG promoter is due to inefficient promoter opening/stabilization steps, (ii) the A/C mismatch has higher transcription efficiency than T/C or C/C. Note that to stably bind the initial NTPs, the +1+2 base pairs need to be fully opened (promoter opening) and kept in a stable opened conformation (stabilization). By monitoring the end-product, that is 2-mer synthesis, we cannot differentiate between defect in promoter opening or stabilization.

When the +1 A:T is pre-melted by C/T mismatch, we observe higher activity (15-fold) with +2 A:T versus G:C (Supplementary Figure S2E). Thus, the identity of +2 base pair appears to dictate the efficiency of transcription initiation by affecting promoter opening/stabilization and 2-mer synthesis.

Similar results were obtained from 2 to 8 mer synthesis with the +2 T:A promoter (Figure 3E). The base-paired +2 T:A promoter has lower *k*_cat_/*K*_m_ of 6 M^−1^ s^−1^ relative to the +2 A:T promoter (Figure 3E and C, asterisk, and Table 1). However, pre-melting the +2 T:A to A/A mismatch both decreases the NTPs *K_m_* to 380 μM and increases the *k*_cat_ to 0.3 s^−1^ (Figure 3E and Table 1, +2 T:A marked by asterisk, and Supplementary Figure S2D). Thus, the *k*_cat_/*K*_m_ of +2 A/A (790 M^−1^ s^−1^) is slightly better than the consensus +2 A:T promoter (630 M^−1^ s^−1^). Changing the +2T to G or C, to create +2 G/A or C/A mismatches, respectively, also lowers the NTPs *K*_m_, but the *k*_cat_ is not increased relative to the +2 T:A promoter (Figure 3E and Table 1). In this series of promoters, clearly the +2 A/A has the highest efficiency. These results indicate that the lower efficiency of transcription initiation on the +2 T:A promoter is also because of inefficient promoter opening/stabilization.

### Promoters with non-template +2A are highly efficient

Although stability of the base pair may play a role in promoter opening, it appears that the transcription efficiency depends on the particular base in the non-template +2 position. For example, in case of AG and AT promoters, mismatches increase activity, but those with AA in the non-template have the highest activity (Figure 3D and E, first bars). This indicates that specific interactions of Rpo41-Mtf1 with the +2 non-template base may play a role in promoter opening/stabilization. A special role of non-template +2A is supported by additional observations with the AA promoter, where pre-melting the +2 A:T to T/T, G/T and C/T mismatches does not substantially increase transcription initiation (1.2–1.7 times) (Figure 3C and Table 1, Supplementary Figure S2B). This indicates that the advantage gained from pre-melting the +2 A:T is balanced out by the loss of +2 non-template A.

### Rpo41-Mtf1 prefers pyrimidines in the +2 template position or purine NTPs

To determine if Rpo41-Mtf1 shows preference for +2 template base, we compared the mismatched promoters with +1+2 AT non-template bases and C or T templating +2 base (Figure 3C and D, second bars). We find that promoter with +2 T template base is more active than +2 C. Similarly, when we compare mismatched promoters with AG as the non-template, and T or A as the templating base, we again find that the promoter with +2 T in the template is more active than +2 A (Figure 3C and E, third bars). Likewise, when we compare mismatched promoters with AC non-template bases, the initiation efficiency is highest with template +2 T, followed by C, and least when it is A (Figure 3A, B and C, fourth bars). These results indicate that Rpo41-Mtf1 prefers T as the +2 template base, followed by +2 C, but +2 A in the template strand is not favorable. This could be due to preference for pyrimidine in the +2 template position and/or specific interactions with the incoming +2 purines ATP or GTP.

There is an exception, however, and that is when the non-template bases are AA (Figure 3D and E, first bar). In the AA promoter, template +2C and +2A are both highly active, indicating thus a special role of the +2 non-template A.

### Higher amounts of 2-mer during initial transcription from promoters with AA sequence

We analyzed RNA products from 2-mer to 8-mer in promoters with all combination of matched and mismatched bases at the +2 position (Figure 4A). The promoters are grouped according to their +1+2 template sequence. For example, promoters with +1+2 template TT that all initiate with ATP and contain A, T, G or C as the +2 non-template base are in one group. In each group, the completely duplexed promoters are marked with an asterisk. As seen in the gel, Rpo41-Mtf1 transcribes well on AA/TT promoter and synthesizes ∼10 μM of 2–8-mer RNA (Figure 4A, lane 2, and 4B, first bar). Consistent with the transcription efficiency trend of natural promoters, changing the +2 A:T to G:C reduces RNA synthesis about 3-fold, but changing to C:G or T:A reduces it further by 12–14-fold (Figure 4A, lanes 2, 8, 13, 15 and 4B, marked with asterisk). Pre-melting the +2 G:C, C:G or T:A base pairs by mutating the +2 non-template base leads to an increase in the total RNA synthesis (Figure 4A, lanes 6–17, and 4B, groups 2–4), however we do not see a similar increase on pre-melting the +2 A:T bp (Figure 4A, lanes 2–5, and 4B, first group). Moreover, we observe that in each group, promoters that show the highest activity are the ones with non-template AA (Figure 4B, bars with arrows). These assays measure 2–8 mer RNA synthesis and therefore are not exactly comparable to the 2-mer synthesis rate experiments (Figure 4B and Table 1). However, the overall trend is consistent with the 2-mer synthesis data and confirms a special role of the +2 non-template A.

Interestingly, promoters with non-template +2A produce relatively high amounts of 2-mer, irrespective of whether the +2A base is matched or mismatched with the template base (Figure 4A, compare lanes 2, 6, 10 and 14 with the remaining three lanes in the respective group). Although the exact reason for higher 2-mer is not known, we postulate that specific interactions of Rpo41-Mtf1 with the non-template +2A aids promoter opening/stabilization to increase 2-mer synthesis, but it may also inhibit efficient translocation of the complex to extend the 2-mer to 3-mer. This would lead to greater fraction of 2-mer accumulating as abortive product.

Another observation from transcription of various 20 base-pair promoters is that Rpo41-Mtf1 makes less 3–6-mer abortive products when the RNAs contain a G:C or C:G base pair at +2 as opposed to T:A or A:U base pair (Figure 4A, lanes 6–13 versus lanes 2–5 and 14–17). We had observed similar behavior on longer promoters with the natural promoter sequence (Figure 1B), where promoters with G:C base pair at +2 showed little abortive products. This re-enforces the idea that having a +2 G:C or C:G base pair results in efficient extension of short RNAs and less accumulation as abortive products, perhaps because of the more stable initially transcribing complexes.

### Importance of the +1 A:T base pair for transcription initiation

Next, we systematically mutated the +1 A:T to matched and mismatched pairs, keeping the +2 A:T constant (Figure 4C). In these transcription assays, we add 250 μM of all NTPs + [γ-^32^P] ATP (for AA promoter) or [α-^32^P] ATP (for the rest) and monitor synthesis of 2-mer to 8-mer on the sequencing gel (Figure 4C). The transcription gel is arranged in a modular fashion, where promoters with the same +1+2 template sequence, i.e. TT, CT, GT and AT, are grouped together, and in each group is shown systematically the change of the +1 non-template base to A, T, G and C. The completely duplexed promoters are marked with an asterisk (Figure 4C, lanes 2, 8, 13 and 15).

Pre-melting the +1 A:T to T/T, G/T and C/T mismatches has no significant effect on transcription activity (Figure 4C, lanes 2–5, and Figure 4D, first group set), similar to the observation with the +2 A:T. Changing +1 A:T to G:C base pair, however, decreases transcription initiation by 7-fold (Figure 4C, lane 2 versus 8), but introducing A/C, T/C or C/C mismatch (Figure 4C, lanes 6–9, and Figure 4D, second group set) restores transcription to the level observed with the matched +1 A:T promoter. Although visually the amount of RNA products from AG promoters appears to be higher, note that the high intensity of the bands is due to multiple [α-^32^P]AMP incorporated in the RNA, as opposed to RNA from the AA promoters that are end-labeled with [γ-^32^P] ATP (Figure 4C, lanes 2–5 versus 6–9), which we normalize in the quantitations. These results indicate that Rpo41-Mtf1 can use promoters that initiate with +1 GTP, as long as the +1 base pair is pre-melted.

Promoters that have purines in the template position at +1 (or initiate with pyrimidine +1 NTP) have low activity. Thus, both +1 C:G and T:A promoters are ∼15-fold less active than +1 A:T (Figure 4C, lanes 2, 13 and 15). In this case, pre-melting +1 C:G base pair to A/G, T/G and G/G mismatches increases activity 5–7-fold (Figure 4C, lanes 10–13, and Figure 4D, third group set), but the activity is not restored to the level observed with +1 A:T. Similarly, changing the +1 T:A bp to A/A, G/A and C/A mismatches increases activity 3–4-fold (Figure 4C, lanes 14–17, and Figure 4D, fourth group set), but again activity compared to +1 A:T is low. This indicates that specific interactions with the +1 pyrimidine template and/or +1 purine NTPs are required for efficient transcription.

Comparison of transcription from promoter changes at the +1 and +2 positions brings out similarities and differences in the requirement of bases at the two positions. At both promoters, pre-melting the base pair increases transcription efficiency, but the identity of the bases is important. At the +2 position, transcription is efficient with all +2 NTPs as long as the +2 base pair is pre-melted, which is in contrast to the +1 position that shows poor initiation with CTP and UTP in spite of pre-melting +1. Transcription is more efficient with adenine at +2, irrespective of whether it is paired or mispaired, which is not the case with the +1 position.

### Importance of the 6-amino group of the non-template AA

**Figure 5.**
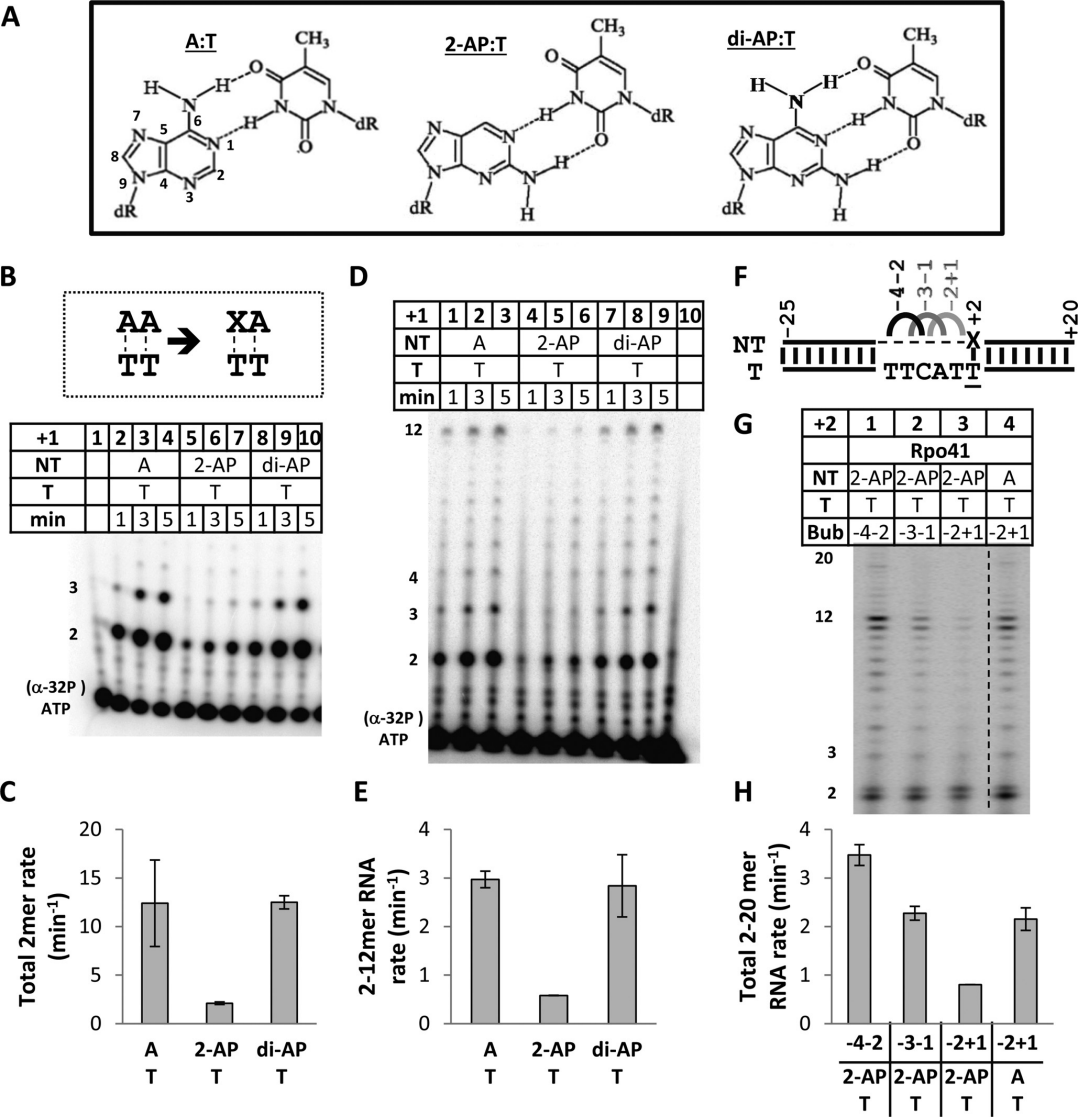
Substitution of +1 and +2 non-template with adenine analogs. (**A**) Adenine, 2-aminopurine and 2,6-diaminopurine structures and base pairing with thymine. (**B**) Time course of 2-mer RNA synthesis from promoters with 2-AP or di-AP at the +1 non-template. The reactions were carried out with 1 μM Rpo41, 2 μM Mtf1 and 2 μM of U25D20 duplex DNA reacted with 250 μM of ATP (+ [α-^32^P] ATP) for 1–5 min. Lane 1, [α-^32^P] ATP alone. (**C**) Rate of total RNA synthesis for each promoter. Standard errors from the linear fitting of data are shown. (**D**) Time course of 2–12 mer RNA synthesis from promoters with 2-AP and di-AP. Reactions' conditions are similar to (B) except 250 μM each of ATP (+ [α-^32^P] ATP), UTP and 3’dGTP was added for 1–5 min. Lane 10, [α-^32^P] ATP alone. (**E**) Rate of 2–12 mer RNA synthesis plotted for each promoter. (**F**) The 3-nt bubble promoters on U25D20 fragment of the 15S rRNA promoter were created by changing the non-template bases; −4–2: GCA (black), −3–1: CAG (dark gray), −2+1: AGC (light gray). The +2 base pair is either A:T or 2-AP:T. (**G**) Transcription gel showing 2–12 mer RNA synthesis on the 3-nt bubble promoters. Reactions were carried out with Rpo41 (3 μM) and DNA (8 μM) in the presence of 200 μM each of ATP (+ [γ-^32^P]), UTP and 3’dGTP for 3 min at 25°C. (**H**) Rates of total 2–20 mer RNA synthesis (moles per mole of enzyme-DNA complex) in the presence of 200 μM each of ATP (+ [γ-^32^P]), UTP and GTP calculated from time courses.

The special role of non-template AA was further investigated by substituting the adenine bases with analogs such as 2-aminopurine (2-AP) that lacks the 6-amino group of adenine, but contains 2-amino group to form a canonical base pair with thymine, and di-AP that contains 2- and 6-amino groups (Figure 5A). The 2-mer (+3-mer from slippage or misincorporation) synthesis rate on the AA promoter is 12.4 min^−1^ (Figure 5B and C), and when we substitute +1 A with 2-AP, the efficiency drops by 6-fold at 2 min^−1^ (Figure 5B, lanes 5–7 and 5C). Interestingly, when we substitute +1 A with 2,6-diaminopurine (di-AP), RNA synthesis rate is restored to 12.5 min^−1^, in spite of Rpo41-Mtf1 having to melt three hydrogen bonds between di-AP:T (Figure 5B, lanes 8–10 and 5C). Similar results are obtained when we monitor 2–12 mer RNA synthesis (Figure 5D). The rate with +1 A and di-AP is 3 min^−1^ and with +1 2-AP is 5-fold lower at 0.6 min^−1^ (Figure 5E). The results indicate that there are specific interactions of Rpo41-Mtf1 with the 6-amino group of the +1 non-template adenine that are probably required for promoter opening/stabilization.

It was reported that Rpo41-Mtf1 efficiently transcribes a duplex promoter with 2-AP base at the +2 non-template position (12). Rpo41 alone can transcribe on 3-nt bubbles in the −4 to +1 region, when +2 is A:T (10), and we employ this as a tool to determine if the +2A specificity is due to interactions with Rpo41. We substitute +2 A:T with 2-AP:T in context of various 3-nt bubbles created by mutating the non-template region between −4 and +1 (Figure 5F) and monitor 2–12 mer RNA synthesis activity of Rpo41 (Figure 5G). On an upstream −4–2 bubble with 2-AP at +2, Rpo41 alone can melt the downstream −1 to +2 region and initiate transcription efficiently (3.5 min^−1^, Figure 5G, lane 1, and 5H) (time course data not shown). However, transcription activity of Rpo41 on +2 2-AP promoters decreases progressively as the melted bubble region is shifted downstream to −3–1 (2.3 min^−1^) and −2+1 (0.8 min^−1^) (Figure 5G, lanes 2–3, and 5H). The transcription activity of Rpo41 on the −2+1 bubble is 3-fold higher when +2 is A:T (2.2 min^−1^) versus 2-AP:T (Figure 5G, lanes 3 and 4, and 5H). These results indicate that Rpo41 requires interactions with the 6-amino group of non-template +2 A, perhaps to melt the −4 and −3 base pairs in the absence of Mtf1. When Mtf1 is present, as reported earlier (12), or the −4 to −2 region is pre-melted, then the requirement of the 6-amino group of +2A becomes less important.

### Rpo41 and Mtf1 are both involved in +2 base-pair specificity

**Figure 6.**
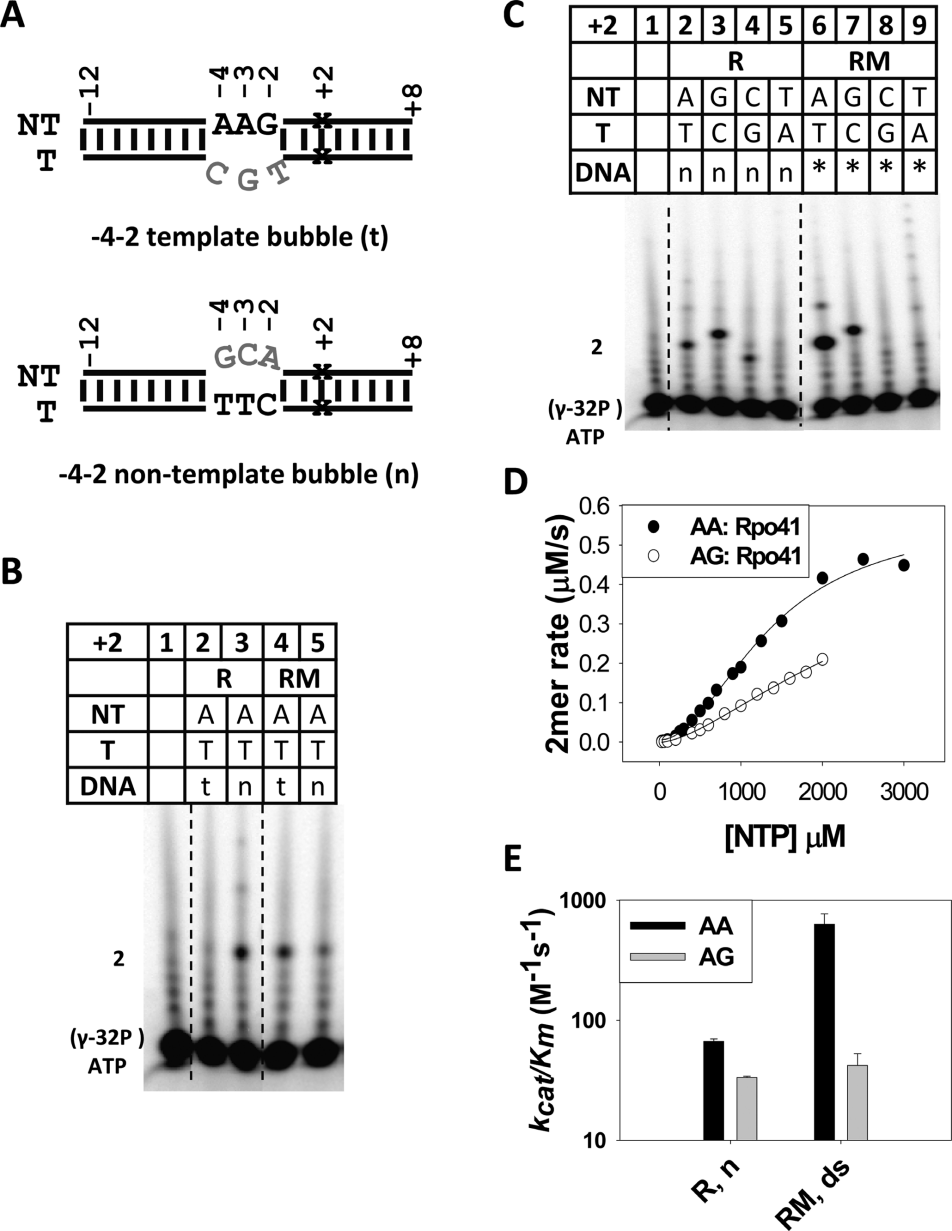
Contribution of Rpo41 and Rpo41-Mtf1 to +2 base-pair specificity. (**A**) The structure of U12D8 bubble promoters; the +2 base pair is either A:T, G:C, C:G or T:A. (**B**) 2-mer synthesis on the +2 A:T bubble promoters (-4–2) with altered template (t) or non-template (n) sequence. The reactions were carried out using 3 μM Rpo41 (R) or Rpo41-Mtf1 (RM), DNA (4 μM) for 5 min at 25°C in the presence of 250 μM ATP (+ [γ-^32^P]). Lane 1, [γ-^32^P] ATP alone. (**C**) 2-mer synthesis by Rpo41 alone on the −4–2 non-template bubbles (n) and Rpo41-Mtf1 on duplex promoters (*) with AA, AG, AT and AC initiation sequences. Transcription was carried out the same as in (B) with 250 μM each of +1 ATP and varying +2 NTP. The amount of 2-mer (AA or AG) appears to be similar, because of the low NTPs concentrations of NTPs used. As seen in (D), the differences become apparent at higher NTP concentrations. (**D**) 2-mer synthesis by Rpo41 alone was measured at increasing NTPs concentration to determine the *k*_cat_/*K*_m_. The reactions contained 4 μM −4–2 non-template bubble promoters, 3 μM Rpo41 and 5–3000 μM ATP (+ [γ-^32^P]) or 5–2000 μM of ATP+GTP (+ [γ-^32^P] ATP). The data are fit to the Hill equation, which provided *k*_cat_ of 0.19 ± 0.013 s^−1^, *K_m_*(ATP) of 1350 ± 110 μM and Hill coefficient of 1.9 for −4–2 bubble with +1+2 AA, and *k*_cat_ of 0.17 ± 0.05 s^−1^, *K_m_*(ATP+GTP) of 2543 ± 762 μM, and Hill coefficient of 1.6 for the bubble with AG. (**E**) Comparison of *k*_cat_/*K_m_* of Rpo41 alone on −4–2 bubble promoters with Rpo41-Mtf1 on duplex promoters (from Figure 3D).

The following experiments were designed to determine whether transcription by Rpo41 itself is more efficient with +2 A:T versus +2 G:C and T:A base pairs. To measure the activity of Rpo41 alone, we use the U12D8 bubble promoters with mismatches in the −4 to −2 region (Figure 6A). There are two ways to make this 3-nt bubble promoter, one where the template sequence in this region is changed, and two where the non-template sequence is changed. Interestingly, Rpo41 does not make 2-mer when the template sequence is altered, although addition of Mtf1 restores activity (Figure 6B, lane 2 versus 4). In contrast, when the −4 to −2 non-template sequence is altered, as observed earlier in Figure 5G, Rpo41 by itself can melt the downstream −1 to +2 region and synthesize 2-mer RNA, but addition of Mtf1 inhibits transcription (Figure 6B, lane 3 versus 5). This is new information that indicates that Rpo41 requires a consensus −4 to −2 template sequence whereas Mtf1 requires a consensus −4 to −2 non-template sequence to initiate efficient transcription.

Using the −4 to −2 bubble promoter where the non-template sequence is changed, we show that Rpo41 by itself is not very specific and synthesizes 2-mer on +2 A:T, +2 G:C and +2 C:G, but not +2 T:A (Figure 6C, lanes 2–5). The catalytic efficiency (*k*_cat_/*K*_m_) of 2-mer synthesis by Rpo41 on the bubble promoter with +2 A:T (141 M^−1^ s^−1^) is only 2-fold higher than +2 G:C (67 M^−1^ s^−1^) (Figure 6D and E). On the other hand, on duplex promoters, Rpo41-Mtf1 is highly specific for +2 A:T and initiates weakly from +2 G:C and +2 T:A (Figure 6C, lanes 6–9). Rpo41-Mtf1 has 16-fold higher specificity for +2 A:T (630 M^−1^ s^−1^) over G:C (40 M^−1^ s^−1^) on the duplex promoter (Table 1 and Figure 6E). The higher specificity of Rpo41-Mtf1 arises from increased *k*_cat_/*K*_m_ of +2 A:T promoter (*k*_cat_/*K*_m_ of 141 M^−1^ s^−1^ for Rpo41 versus 630 M^−1^s^−1^ for Rpo41-Mtf1) rather than lowered *k*_cat_/*K*_m_ of +2 G:C (*k*_cat_/*K*_m_ of 67 M^−1^ s^−1^ for Rpo41 versus 40 M^−1^ s^−1^ for Rpo41-Mtf1). It should be noted that catalytic efficiencies of Rpo41 and Rpo41-Mtf1 have been tested on different DNA substrates, and hence their absolute values are not directly comparable; however, the specificity or the ratio of the catalytic efficiencies of AA and AG for duplex (16-fold) versus bubble (2-fold) should be comparable. Thus, the increased preference for AA in the presence of Mtf1 is either because of direct interactions of Rpo41-Mtf1 with the +1+2 template/non-template region that regulate melting of +2 A:T bp, or by an indirect mechanism, where Rpo41-Mtf1 melts the −4 to −2 region efficiently to create a competent open complex, which is not achieved by Rpo41 alone even when the −4 to −2 is pre-melted.

Taken together, these results indicate that differential promoter efficiencies are achieved by selectively augmenting the catalytic initiation efficiencies of promoters with +2 A:T, and both Rpo41 and Mtf1 contribute to specificity for the +2 A:T in the promoter sequence and/or specificity for initiation with +2 ATP.

## DISCUSSION

We have investigated the biochemical basis of the higher transcription initiation efficiency of the yeast mitochondrial RNA polymerase Rpo41-Mtf1 complex on promoters that initiate with the +1+2 AA sequence. The mitochondrial promoters of the yeast have a consensus −8 to +1 sequence, but initiate with +1+2 AA, AG or AT coding sequence. By studying the complete transcription profile of five representative promoters, we show that AA promoters produce the highest amount of run-off product, followed by +2 G:C, and least efficient is +2 T:A. Although, +1+2 AA promoters are highly efficient, they produce ∼5–10-fold more abortive products than the AG promoters. One explanation for the lower amount of abortive products on AG promoters is the stability of short initial RNA products bound to the RNAP–DNA complex. This is further exemplified by the difference in the abortive products of the two AA promoters, where Cox1 with a +5 G:C base pair synthesizes ∼3-fold less abortive products than the 15S rRNA promoter with no such G:C base pair. Consistent with this, we observe accumulation of 2-mer to 4-mer abortive products on Cox1 promoter, after which they significantly reduce.

Interestingly, the melting/unstacking of the upstream −4 and −1 base pairs does not depend on the identity of the +1+2 base pairs, but functional assays indicate that the sequence of +1+2 base pairs affects the transcription initiation efficiencies. To understand how +1+2 template and non-template bases regulate transcription initiation, we carried out a systematic study where we changed the +1+2 base pairs to all possible paired and mispaired combinations and assessed transcription initiation efficiency from the *k*_cat_/*K_m_* of 2-mer synthesis on short 20 bp promoters that have been successfully used previously to study the initial DNA binding, bending and melting steps (7,11). Consistent with the trends on the natural promoters, we observed that the catalytic efficiency of 2-mer synthesis on the 20 bp promoters is highest for promoters initiating with AA, ∼16-fold lower for AG and ∼105-fold lower for AT. Introduction of single mismatches increases transcription efficiencies, which indicates that transcription initiation efficiency is rate-limited by downstream +1+2 promoter melting, which is required to form the pre-transcribing complex that binds the initiating NTPs. We propose that the lower efficiency of downstream promoter melting with +1+2 AG and TA promoters, but not AA, is responsible for the increased +1+2 NTPs *K*_m_ and lower *k*_cat_ of 2-mer synthesis.

Changing the +1 A:T base pair to any other base pair lowers the initiation efficiency. Even a small change such as substitution of the +1 A:T to 2-AP:T inhibits transcription, which indicates that Rpo41-Mtf1 has specific interactions with the 6-amino group of +1 A in the non-template strand, which is important for efficient downstream promoter melting. Promoter with +1 G:C initiates poorly and pre-melting it by changing the +1 non-template G to A, T or C increases transcription to the level observed with the AA promoter. On the other hand, promoters with +1 T:A and +1 C:A initiate poorly, but pre-melting by changing the +1 non-template to alternative bases does not increase the activity to the level observed with the AA promoter. Thus Rpo41-Mtf1 initiates efficiently when the +1 NTP is purine.

For the +2 position, changing the +2 A:T to any other base pair decreases transcription, and pre-melting the +2 base pair restores or increases transcription efficiency to a greater extent than observed with the AA promoter (Figure 5A and B). In contrast to the +1 position, however, the identity of the +2 non-template base in the mismatch is important and promoters with non-template +2 A have higher activity. If the +1+2 non-template bases are not AA in the mismatches, then initiation occurs efficiently only with purine +2 NTPs/pyrimidine template. However, if the +1+2 is AA, then initiation is efficient even with a pyrimidine +2 NTP/purine template. Again the major effect is on the observed *K_m_* of the +1+2 NTPs, which we propose is due to inefficient conversion of the ‘open complex’ to ‘pre-transcribing complex’. The +1+2 NTPs binding stabilizes the pre-transcribing complex, and if the equilibrium is unfavorable as in +2 G:C or T:A promoters, higher concentrations of +1+2 NTPs are required to drive the reaction forward resulting in higher observed *K_m_* of the +1+2 NTPs. A similar mechanism exists in the homologous T7 RNAP, where binding of the +2 NTP is required to favorably shift the equilibrium from a closed complex to an open complex (21).

It was proposed that mitochondrial RNAP acts as an ATP sensor *in vivo* and regulates the transcript abundance in accordance with the varying ATP pools during respiration and fermentation (1,18). However, another study showed that the *K_m_* of +2 NTP is higher than the *K_m_* +1 ATP (19), which would suggest that transcription initiation is limited by the binding efficiency of +2 NTP rather than +1 ATP, especially because the *in vivo* ATP levels in the mitochondria of mammalian tissues are highest (2.3–8 mM), followed by GTP (0.2–1.3 mM), and lowest for CTP (<0.01–0.24) and UTP (0.01–0.13) (22,23). In accordance, this study also shows that transcription efficiency of natural promoters is primarily regulated by their varying +2 sequence, affecting the composite *k*_cat_/*K_m_* of +1+2 NTPs rather than just *K_m_* ATP. Steady-state transcript abundance in cells on the other hand is influenced not only by the rate of RNA synthesis but also by its degradation rate, splicing and other post-transcriptional processing of transcripts, regulation by nuclear factors and the nutrient status of the cell (21,24–27); what precise steps are modulated by the varying ATP levels remains to be determined.

**Figure 7.**
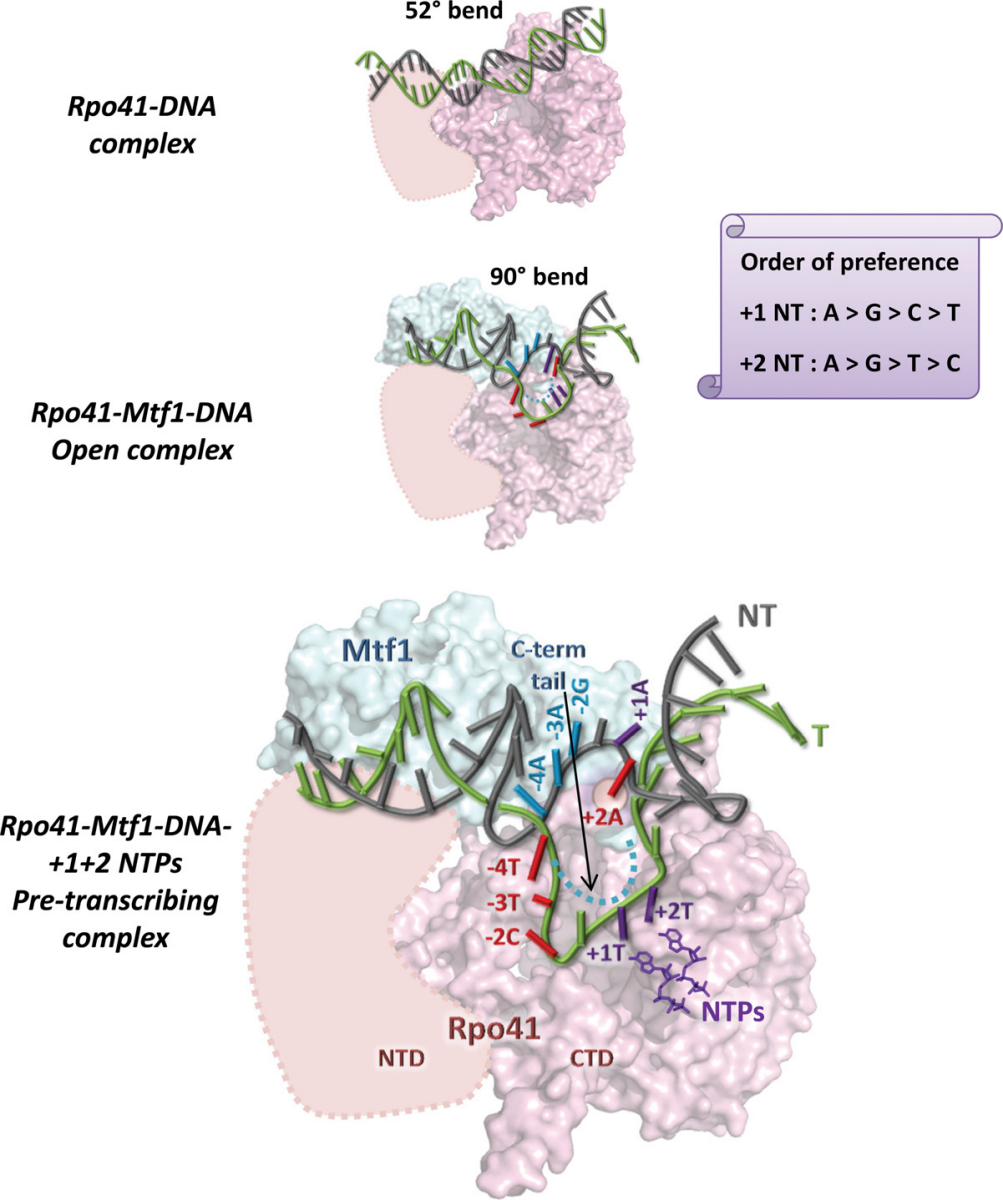
Model of transcription initiation by the yeast mitochondrial RNA polymerase. Rpo41 (pink) with the conserved C-terminal catalytic domain (416–1217) model is based on its homolog T7 RNAP (1,9), and the N-terminal domain is outlined (pink dotted). The orientation of Mtf1 (PDB code 1I4W) and its C-terminal tail (blue dotted) is speculative, but its positioning with respect to the DNA is based on previous crosslinking studies (9,10) and crystal structures of homologous transcription initiation and elongation complexes (26,28–30). The figure shows three intermediate stages during formation of a competent pre-transcribing complex, each with varying degrees of unstacking in the −4 to +2 region of DNA (template: green, non-template: gray). Top: Rpo41 alone binds to the promoter DNA and induces a 52° bend without melting the DNA. Middle: Rpo41-Mtf1 induces a severe 90° bend and melting in the promoter DNA with major unstacking of −4 and −3 bases (open complex). Bottom: Binding of +1 and +2 NTPs (purple) results in unstacking of the −1 to +2 region and stabilization of the melted +1+2 template bases in Rpo41's active site for efficient catalysis (pre-transcribing complex). Specific interactions of Rpo41 with the −4 to −2 template bases are indicated in red, and those of Mtf1 with the −4 to −2 non-template bases are in blue. Interactions of Rpo41-Mtf1 with the +1 and +2 template and non-template bases and the initiating NTPs are shown in purple. Rpo41 specifically interacts with the 6-amino group of +2 non-template adenine (red) to regulate promoter initiation efficiency. The preference of Rpo41-Mtf1 for purines at +1 and +2 non-template positions is indicated on the scroll.

Based on available data, we postulate that the yeast mitochondrial initiation complex shows three intermediates: (i) Rpo41-DNA complex, (ii) Rpo41-Mtf1-DNA *open complex* and (iii) Rpo41-Mtf1-DNA-+1+2NTPs *pre-transcribing complex* (Figure 7), and the +1+2 promoter sequence modulates RNA synthesis efficiency by specifically affecting formation of a competent pre-transcribing complex (discussed below). The relative positioning of Rpo41 (with its conserved C-terminal catalytic domain modeled based on homology with T7 RNAP) (1,9), Mtf1 (PDB code 1I4W), and DNA in the intermediates is based on previous cross-linking studies (9,10), and crystal structures of homologous transcription initiation and elongation complexes (26,28–30).

Previous studies have shown that Rpo41 has tight interactions with the promoter (*K*_d_ ∼58 nM) and Rpo41 by itself bends the promoter by 52° and shows transient visitations to the severely bent DNA conformation (7,11). Our 2-AP bubble data indicates that Rpo41 by itself can melt the downstream −1 to +2 promoter region and initiate efficient transcription when the upstream −4 to −2 region is pre-melted; however, it has trouble melting the upstream −4 and −3 base pairs. Addition of Mtf1 increases the visitations to the severely 90° bent DNA conformation, and 2-AP experiments show that the promoter in the Rpo41-Mtf1 complex is melted from the −4 to +2 region, with prominent unstacking of −4 and −3 bases (7,12). Together with the strong Mtf1 crosslinks observed in the −4 to −2 promoter region (9,10), these studies suggest that Mtf1 stabilizes the bent/melted DNA conformation, probably by stably melting the upstream −4/−3/−2 region. Consistent with this, our studies also indicate that sequence specific interactions of the non-template bases with Mtf1 and template bases with Rpo41 in the −4 to −2 region are essential for efficient transcription initiation. These interactions of Rpo41-Mtf1 likely aid upstream promoter DNA melting from −4 to −2/−1 to form the bent/melted ‘open complex’; however, we propose that the +1+2 base pairs are not stably melted in this ‘open complex’.

Our results indicate that initiating NTPs are required to stably unstack the −1 template base and effectively melt/stabilize the +1+2 region to generate the ‘pre-transcribing complex’, where the template bases are optimally aligned in Rpo41's active site to facilitate correct start-site selection and 2-mer synthesis. In addition to multiple interactions throughout the −4 to +2 region, specific interactions with the 6-amino groups of +1 and +2 non-template adenines play an important role in stabilization of pre-transcribing complex and efficient transcription from the AA promoter.

Unlike the extensive non-template interactions in the yeast mitochondrial initiation complex, the homologous T7 RNAP does not have any significant interactions with the melted non-template strand during initiation (28,31), indicating an evolutionary divergent mechanism of melting in yeast mitochondria where Mtf1 traps the melted non-template strand and stabilizes the open complex. In addition to melting, Mtf1 also plays a significant role in start-site selection (10) and in +2 base-pair specificity. Our studies show that Rpo41 has some intrinsic preference for initiating on promoters with +2 A:T, but the promoter specificity is much heightened in the presence of Mtf1. It remains to be determined whether Mtf1 directly interacts with the +2 A:T base pair or allosterically orients the template bases and/or the initiating NTPs in Rpo41's active site to achieve specific initiation.

Interactions with +2 base are observed in the bacterial initiation complex, where the core RNAP β subunit flips the +2 non-template G base and buries it in a β-pocket (26). Mutating the +2 G to any other base results in 5-fold lower equilibrium dissociation constant and a 5-fold lower off-rate of the DNA, which suggests that the interactions with +2 non-template base are sequence specific. Similar flipping out of +2 non-template base is observed in the yeast RNAP II backtracked and arrested elongation complex (32). Our studies suggesting specific interactions of Rpo41 with +2 A are consistent with other transcriptional systems. If this specificity persists during elongation, it could affect events such as sequence specific pausing, in addition to the pausing phenomenon we observe during initiation that results in excess 2-mer RNA formation whenever the +2 mismatched base is adenine.

Despite great diversity in promoter architecture, RNAP assembly and mechanism of transcription regulation, the yeast mt transcription system seems to share common RNAP–DNA interactions with the bacteriophage, prokaryotic and eukaryotic systems. Almost all single- and multi-subunit RNAPs initiate with purines, hinting at a conserved mechanism of start-site selection. In this paper, we have established the biochemical basis of preference for initiating purines in yeast mitochondria. We envision that the sequence-specific information will facilitate designing of optimal DNA substrates for capturing structural snapshots of the thus-so-far missing initiation complexes of yeast mitochondria. In addition to providing structural and mechanistic insights, this study has potential applications in developing commercial tools for custom RNA synthesis.

## SUPPLEMENTARY DATA

Supplementary Data are available at NAR Online.

SUPPLEMENTARY DATA
