# Supplementary material for: Interactions of the yeast mitochondrial RNA polymerase with the +1 and +2 promoter bases dictate transcription initiation efficiency
Source: Nucleic Acids Res. 2014 Sep 23;42(18):11721–32. doi: 10.1093/nar/gku868 (PMC4191429; doi:10.1093/nar/gku868)
Supplement: SUPPLEMENTARY DATA [file supp_42_18_11721__index.html]

Interactions of the yeast mitochondrial RNA polymerase with the +1 and +2 promoter bases dictate transcription initiation efficiency — Interactions of the yeast mitochondrial RNA polymerase with the +1 and +2 promoter bases dictate transcription initiation efficiency — SUPPLEMENTARY DATA 

# Interactions of the yeast mitochondrial RNA polymerase with the +1 and +2 promoter bases dictate transcription initiation efficiency

## SUPPLEMENTARY DATA

**Files in this Data Supplement:**

- SUPPLEMENTARY DATA
