# Supplementary material for: Interactions of the yeast mitochondrial RNA polymerase with the +1 and +2 promoter bases dictate transcription initiation efficiency
Source: Nucleic Acids Res. 2014 Sep 23;42(18):11721–32. doi: 10.1093/nar/gku868 (PMC4191429; doi:10.1093/nar/gku868)
Supplement: SUPPLEMENTARY DATA [file supp_gku868_nar-01625-h-2014-File010.pdf]

Supplementary information

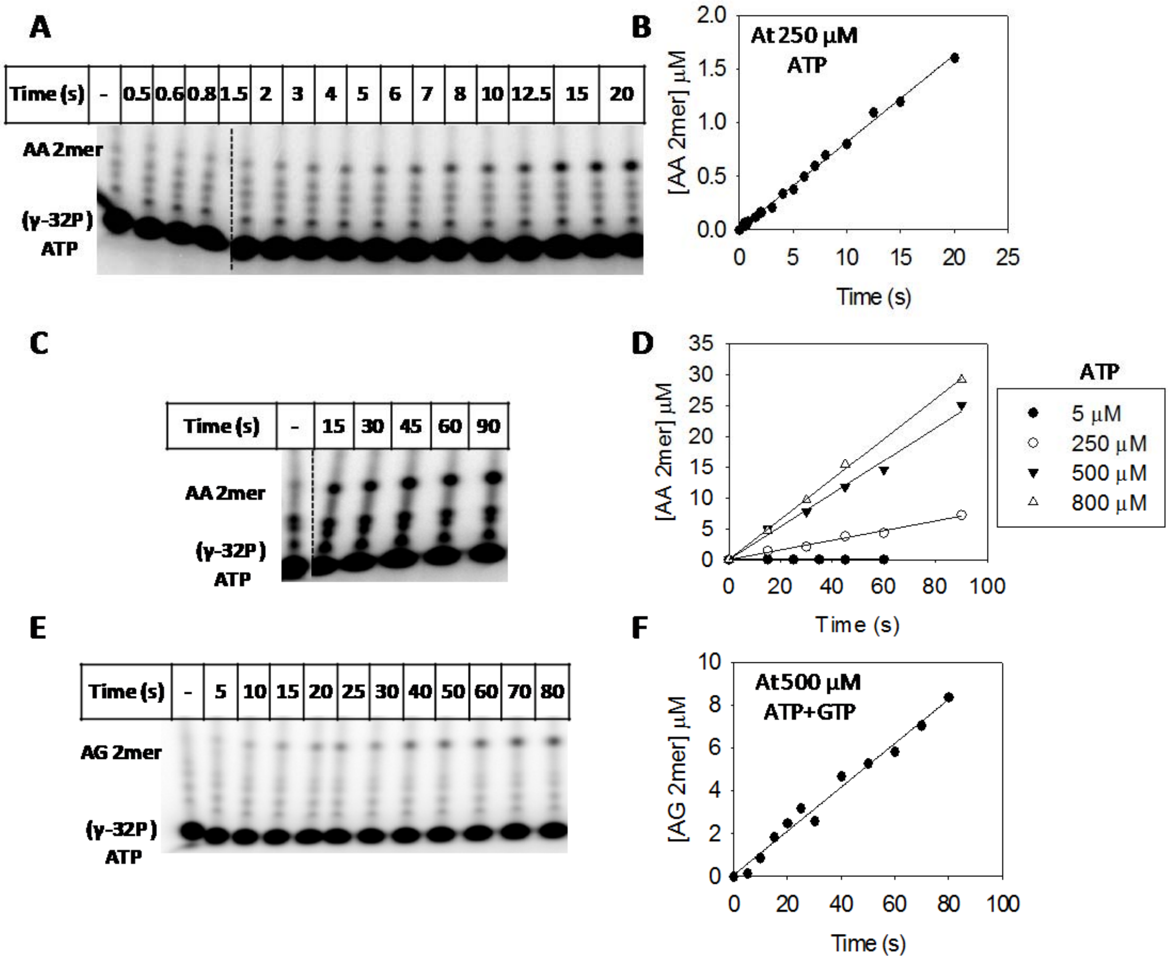

Supplementary Figure S1: Time course of 2-mer RNA synthesis on 15S and 21S rRNA promoters. (A) Transcription gel showing pre-steady state kinetics of AA 2-mer RNA synthesis at 250 μM ATP. A preincubated complex of 2 μM Rpo41, 2.5 μM Mtf1 and 2.5 μM U25D32 15S rRNA (AA/TT) promoter, rapidly mixed with equal volume of 500 μM ATP (+ [γ-^{32}P] ATP) at 25°C in a quenched-flow set-up, and quenched at different time intervals starting from 0.5 s. Note that the final concentration of the reactants would be half of the initial concentrations. (B) The plot of molar amount of AA 2-mer against time fit to linear function and provided a 2mer synthesis rate of 0.075 μM.s^{-1} under pre-steady state conditions. (C) Representative

transcription gel showing AA 2-mer synthesis between 15-90 s. 1 μM Rpo41, 2 μM Mtf1 and 2 μM U12D8 ds 15S rRNA DNA was reacted with 5-1000 μM ATP (+ [γ-^{32}P] ATP) and 2-mer synthesis was monitored at 25 °C. (D) Molar amount of 2-mer RNA synthesized showing linear kinetics at various ATP concentrations. (E) Transcription gel showing pre-steady state kinetics of AG 2-mer RNA synthesis. 8 μM Rpo41, 10 μM Mtf1 and 10 μM U12D8 ds 21S rRNA (AG/TC) promoter was reacted with 500 μM of both ATP (+ [γ-^{32}P] ATP) and GTP, and AG 2mer synthesis was monitored at 25 °C over different time intervals. (F) The plot of molar amount of AG 2-mer against time fit to linear function and provided a 2mer synthesis rate of 0.1 μM.s^{-1} under pre-steady state conditions. The first lanes have control samples with [γ-^{32}P] ATP alone.

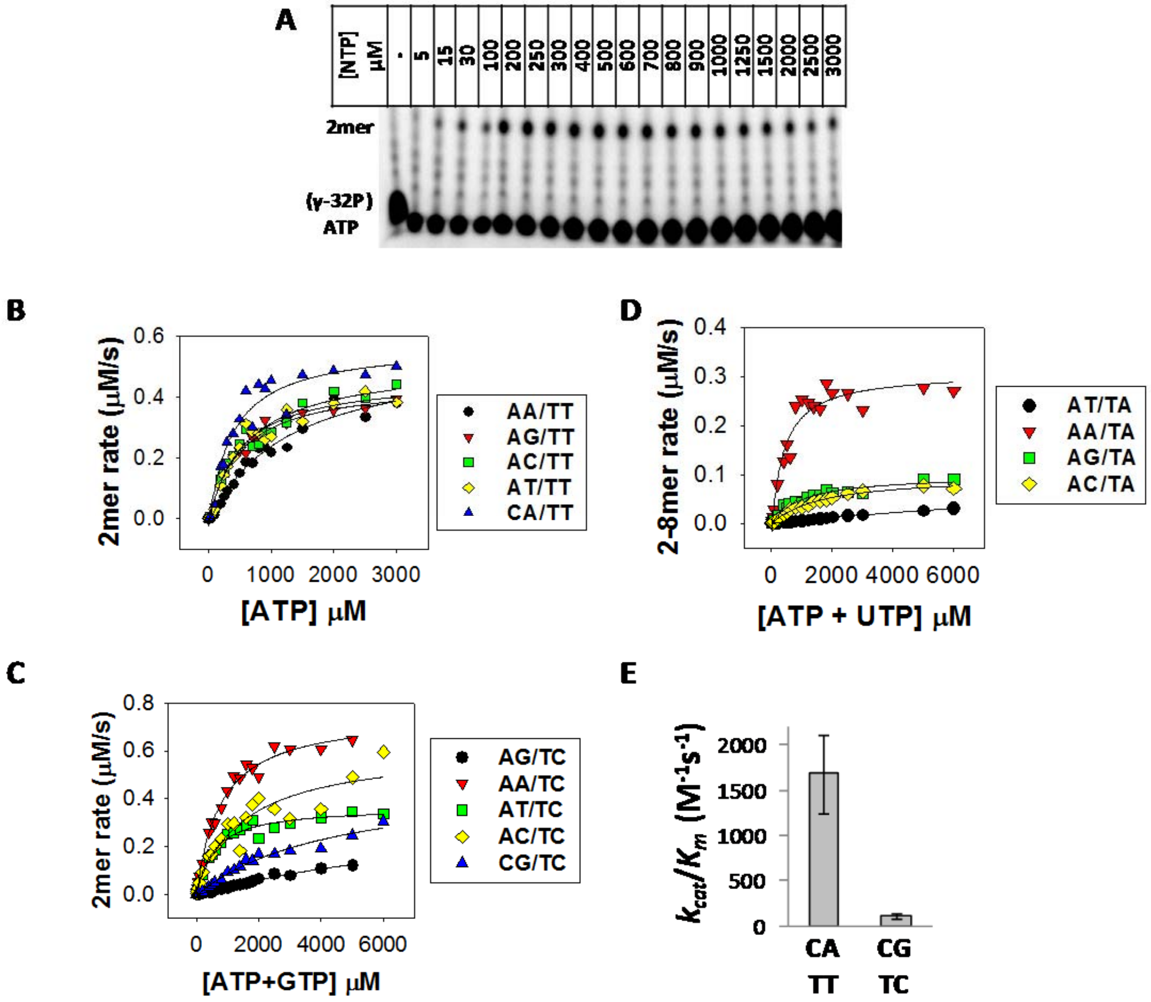

Supplementary Figure S2: Plots of 2-mer synthesis rates for calculating composite catalytic efficiency (*k_{cat}/K_m*) of binding +1 and +2 NTPs. (A) Representative gel showing synthesis of 2-mer RNA as a function of increasing concentration of +1 and +2 NTPs. A pre-incubated complex of 1 µM Rpo41, 2 µM Mtf1 and 2 µM U12D8 ds DNA was reacted with increasing concentration of 5-3000 µM of ATP (+ [γ-^{32}P] ATP) for promoters initiating with AA, or 5-3000 µM each of +1 ATP (+ [γ-^{32}P] ATP) and +2 GTP or +2 UTP for promoters initiating with AG or AU respectively. Plots of 2-mer synthesis rates against increasing concentrations of initiating +1+2 NTPs for promoters with template +1+2 TT (B), +1+2 TC (C) and +1+2 TA (D), and

varying +1 and +2 non-template bases, fit to Michaelis-Menten kinetics. (E) Comparative k_{cat}/K_m ratio plot for promoters with mismatches at +1.
